# Exploring the advances of single-cell RNA sequencing in thyroid cancer: a narrative review

**DOI:** 10.1007/s12032-023-02260-x

**Published:** 2023-12-21

**Authors:** Joecelyn Kirani Tan, Wireko Andrew Awuah, Sakshi Roy, Tomas Ferreira, Arjun Ahluwalia, Saibaba Guggilapu, Mahnoor Javed, Muhammad Mikail Athif Zhafir Asyura, Favour Tope Adebusoye, Krishna Ramamoorthy, Emma Paoletti, Toufik Abdul-Rahman, Olha Prykhodko, Denys Ovechkin

**Affiliations:** 1 Faculty of Medicine, University of St Andrews, St Andrews, Scotland, UK; 2 Faculty of Medicine, Sumy State University, Sumy, Ukraine; 3 School of Medicine, Queen’s University Belfast, Belfast, UK; 4 School of Clinical Medicine, University of Cambridge, Cambridge, UK; 5 Faculty of Medicine, Bangalore Medical College and Research Institute, Bengaluru, India; 6 School of Medicine, The University of Nottingham, Nottingham, NG7 2UH UK; 7 Faculty of Medicine, Universitas Indonesia, Jl. Salemba Raya No.6, Jakarta, 10430 Indonesia; 8 Rutgers University-New Brunswick, New Brunswick, NJ 08854 USA; 9 Faculty of Medicine, University of Manchester, Manchester, M13 9WJ UK

**Keywords:** Single-cell RNA sequencing, Thyroid cancer, Personalised medicine, Tumour microenvironment, Tumour heterogeneity, Medical oncology

## Abstract

Thyroid cancer, a prevalent form of endocrine malignancy, has witnessed a substantial increase in occurrence in recent decades. To gain a comprehensive understanding of thyroid cancer at the single-cell level, this narrative review evaluates the applications of single-cell RNA sequencing (scRNA-seq) in thyroid cancer research. ScRNA-seq has revolutionised the identification and characterisation of distinct cell subpopulations, cell-to-cell communications, and receptor interactions, revealing unprecedented heterogeneity and shedding light on novel biomarkers for therapeutic discovery. These findings aid in the construction of predictive models on disease prognosis and therapeutic efficacy. Altogether, scRNA-seq has deepened our understanding of the tumour microenvironment immunologic insights, informing future studies in the development of effective personalised treatment for patients. Challenges and limitations of scRNA-seq, such as technical biases, financial barriers, and ethical concerns, are discussed. Advancements in computational methods, the advent of artificial intelligence (AI), machine learning (ML), and deep learning (DL), and the importance of single-cell data sharing and collaborative efforts are highlighted. Future directions of scRNA-seq in thyroid cancer research include investigating intra-tumoral heterogeneity, integrating with other omics technologies, exploring the non-coding RNA landscape, and studying rare subtypes. Overall, scRNA-seq has transformed thyroid cancer research and holds immense potential for advancing personalised therapies and improving patient outcomes. Efforts to make this technology more accessible and cost-effective will be crucial to ensuring its widespread utilisation in healthcare.

## Introduction

Thyroid cancer, a prevalent form of endocrine malignancy, has observed a marked increase in incidence over recent decades [1, 2]. In 2020, there were approximately 586,000 new cases of thyroid cancer, constituting 2.5% of all cancer diagnoses [2, 3]. This reflects the increasing burden of thyroid cancer on global health. Contributing factors to thyroid cancer development include overexposure to radiation [4], insufficient or excessive iodine consumption [5], elevated thyroid-stimulating hormone (TSH) levels, mitogenic effects of the insulin/insulin-like growth factor (IGF) system, metabolic and insulin resistance syndromes, and obesity [6].

Cancers of the thyroid are often categorised according to their cellular aetiology, classifying them into papillary, follicular, medullary, and anaplastic types. Approximately 90% of these cancers are papillary and follicular, which are differentiated thyroid carcinomas (DTCs) derived from follicular epithelial cells [7]. Medullary thyroid carcinoma (MTC), another subtype, is a neuroendocrine tumour originating from parafollicular cells. Anaplastic thyroid carcinoma (ATC), a rare subtype representing less than 1% of thyroid cancers, evolves from further differentiation of DTCs [8]. Prognosis varies across these subtypes, with ATC presenting the least favourable survival rate [8, 9].

Thyroid carcinoma frequently manifests as a solitary nodule in the neck, detected either by physical examination or imaging such as computerised tomography (CT) and magnetic resonance imaging (MRI). Once a nodule is discovered, ultrasonography serves as the first-line investigative measure. However, fine-needle aspiration under ultrasound guidance for cytological examination is essential to confirm whether the nodule is malignant or benign. Specific intraoperative tests follow to determine the cancer’s cellular origins [10–12].

As the incidence of thyroid carcinomas rises, attributed in part to environmental pollution and obesity [13, 14], it is critical to expand our understanding of this disease. One promising method is single-cell ribonucleic acid sequencing (scRNA-seq), which offers transcriptome-wide profiling at the single-cell level [15]. This technique provides comprehensive insights into the heterogeneity and complexity of the thyroid tumour microenvironment (TME), revealing the widespread intratumoral variations [15, 16].

ScRNA-seq involves the isolation of individual cells, and the reverse transcription of their RNA to complementary deoxyribonucleic acid (cDNA), followed by amplification and sequencing [16]. This process yields a comprehensive gene expression profile for each cell, exposing the complexity of cell populations within thyroid tumours [16]. High-throughput analysis has become achievable through technologies such as droplet-based microfluidic platforms [17], although challenges persist with technical variances and biases [18]. Nevertheless, progress in computational tools assists in interpreting the large datasets produced by scRNA-seq, allowing for greater insights into cell-specific functions and states in thyroid carcinomas [19]. This progress is key to advancing our understanding and treatment of these increasingly prevalent cancers.

This review seeks to elucidate the use and effectiveness of scRNA-seq in thyroid cancer research. It explores recent advancements in the technique, discusses its limitations, and explains how current research addresses these issues. Ultimately, this review aims to offer an inclusive discussion on the application of scRNA-seq in thyroid cancer research.

## Methodology

This narrative review aims to provide a comprehensive evaluation of the role of scRNA-seq in thyroid cancer research. To ensure a thorough and inclusive analysis, specific inclusion and exclusion criteria were employed.

The inclusion criteria for this review consisted of full-text articles written in English, spanning from 2000 to 2023. This time period was selected to enable a thorough evaluation of established practices within the field and capture any significant advancements over a substantial period. Multiple databases, including PubMed, EMBASE, and Web of Science, were systematically searched to ensure a comprehensive literature base.

Key search terms such as “scRNA Sequencing” and “single-cell sequencing” were used alongside thyroid cancer-specific terms like “thyroid Cancer”, “thyroid tumours”, “canceromics”, “tumour microenvironment”, and “cancer heterogeneity”. This approach ensured the inclusion of relevant articles, focusing on the intersection of scRNA-seq and thyroid cancer, into the review.

In addition to a systematic database search, references cited in recent thyroid cancer reviews have been manually examined to identify additional supplementary sources. Exclusion criteria were applied to omit standalone abstracts, case reports, posters, and unpublished or non-peer-reviewed studies. This approach enabled the prioritisation of high-quality, reliable evidence.

**Fig. 1:**
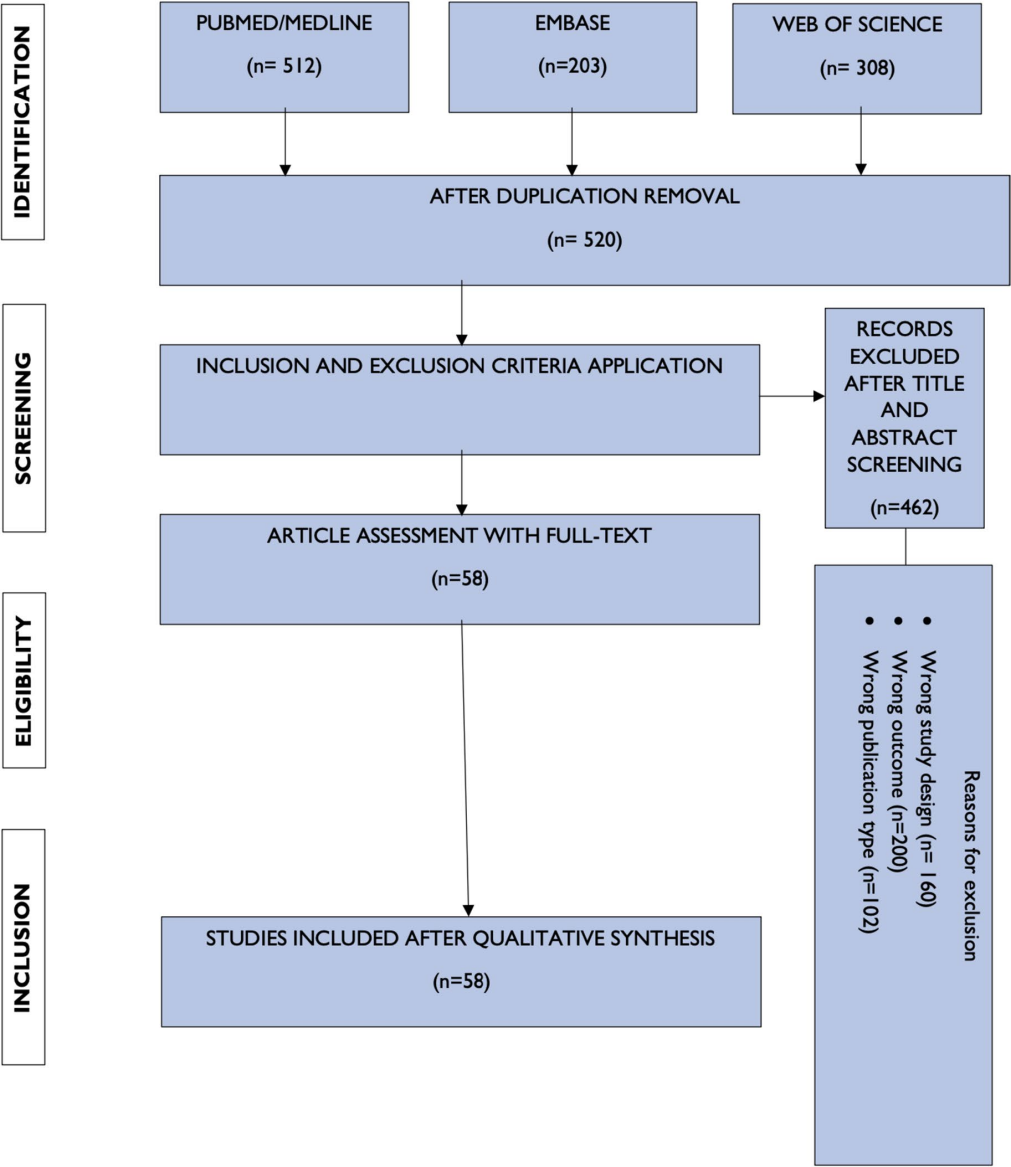
PRISMA flow diagram summarising the methodology used (Image originally created by authors)

The scope of the review did not confine the number of studies to be included, intending to gather a comprehensive understanding of the topic and include a wide array of study designs. The review integrates descriptive studies, animal-model studies, cohort studies, and observational studies, offering a holistic perspective on the application of scRNA-seq in thyroid cancer research. Both pre-clinical and clinical studies were included in order to extend the breadth of knowledge covered in this review. Figure 1 summarises the methodology employed.

## Single-cell RNA sequencing applications and outcomes in thyroid cancer

### Insights into cellular heterogeneity and tumour microenvironment

The tumour ecosystem of papillary thyroid carcinoma (PTC), the most commonly observed thyroid cancer, remains poorly characterised, as does the complex process of intra-tumour transformation in ATC [15, 20–22]. Moreover, the mechanisms by which PTC tumour cells with lymph node (LN) metastasis evade immune surveillance and colonise distant organs remain uncertain [23]. Recent advancements in scRNA-seq technology have unveiled the complex cellular landscape of thyroid cancers, uncovering novel degrees of heterogeneity and potential drivers of recurrence and tumour microenvironments (TMEs) [20].

Detailed scRNA-seq data analysis in PTC reveals an intricate network of cells comprising malignant follicular epithelial cells (MFECs), CD8+ and CD4+ T cells, B cells, vascular endothelial cells, fibroblasts, and cancer-associated fibroblasts (CAFs) [24]. Furthermore, the proportion of normal follicular epithelial cells (NFECs) and interstitial cells was significantly higher in paracancerous tissue (PCa) [24]. Notably, some B cells in primary tumours exhibit inhibitory receptors, with corresponding ligand genes transcribed in T cells and malignant epithelial cell clusters. Meanwhile, certain CD8+ T cells in both primary tumours and LN overexpress inhibitory receptors with matching ligands present in some CD4+ T cells [24].

During the advanced disease stage, a decrease in *IFNG*-expressing CD8+ tissue-resident memory T cells is observed, coupled with an increased ratio of suppressive M2-to-pro-inflammatory M1-like macrophages [25]. Several key biological interactions among myeloid cells, T cells, and follicular cells have been detected, related to T-cell recruitment, M2-like macrophage polarisation, malignant follicular cell progression, and T-cell inhibitory signalling [25]. Of particular interest is the potential regulatory role of fibroblasts in immune cell functions via the Macrophage Migratory Inhibition Factor (MIF) signalling pathway in the TME, promoting thyroid cancer development [15]. In addition, an elevated proportion of CD4+ T cells could contribute to the immunosuppressive characteristics of PTC [26].

Analysis of scRNA-seq data on PTC also reveals a ‘cancer-primed’ premalignant thyrocyte population, displaying normal morphology but altered transcriptomes [20]. The investigation identified three distinct phenotypes, including follicular-like, partial-epithelial-mesenchymal-transition-like, and dedifferentiation-like malignant thyrocytes, each influencing bulk molecule subtypes, tumour traits, and responses to radioactive iodine (RAI) [20]. Additionally, the integration of scRNA-seq-enabled analysis with CellPhoneDB revealed interactions between lymphatic endothelial cells (ECs) and immune cells in PTCs through the atypical chemokine receptor 2 (ACKR2), Intercellular adhesion molecule-1 (ICAM-1), and the critical angiogenic vascular endothelial growth factor (VEGF) and its receptor (VEGFR) signalling [20]. These findings underscore the presence of extensive vascular-immune crosstalk within the multicellular tumour ecosystem, thereby enhancing our understanding of cellular heterogeneity and the TME of thyroid cancer [20].

In the broader context of thyroid cancer, including PTC, MTC, and ATC, scRNA-seq data facilitated the identification of thyroid cell deterioration processes spanning normal, intermediate, and malignant cells [1]. Cell-to-cell communication analysis revealed a strong link between thyroid cells, fibroblasts, and B cells in the MIF signalling pathway [1]. Furthermore, a strong correlation between thyroid cells and B cells, TampNK cells, and bone marrow cells was revealed [1]. These findings deepen our understanding of thyroid cancer’s TME, driving the development of personalised medicine.

Intriguingly, scRNA-seq not only enhances our understanding of the single-cell landscape of human PTC but also the immunological link between PTC and Hashimoto’s thyroiditis (HT). To begin with, intertumoral heterogeneity could be revealed based on the *BRAF V600E* mutation or LN metastasis [27]. Additionally, transcription factor regulons of follicular epithelial cells reveal different transcription activation states in PTC patients with or without concurrent HT [27]. The immune cells in tumours exhibited distinct transcriptional states, and the presence of tumour-infiltrating B lymphocytes was predominantly linked to a concurrent HT origin [27]. Trajectory analysis of B cells and plasma cells suggested their migration potential from adjacent HT tissues to tumour tissues. Finally, the analysis revealed diverse LR pairs between non-immune cells, infiltrating myeloid cells, and lymphocytes [27]. These findings deepen our knowledge of cellular heterogeneity in the PTC microenvironment and present the immunological link between PTC and HT [27]. As such, these findings could inform future studies and efforts to identify novel biomarkers and therapeutic interventions.

**Fig. 2:**
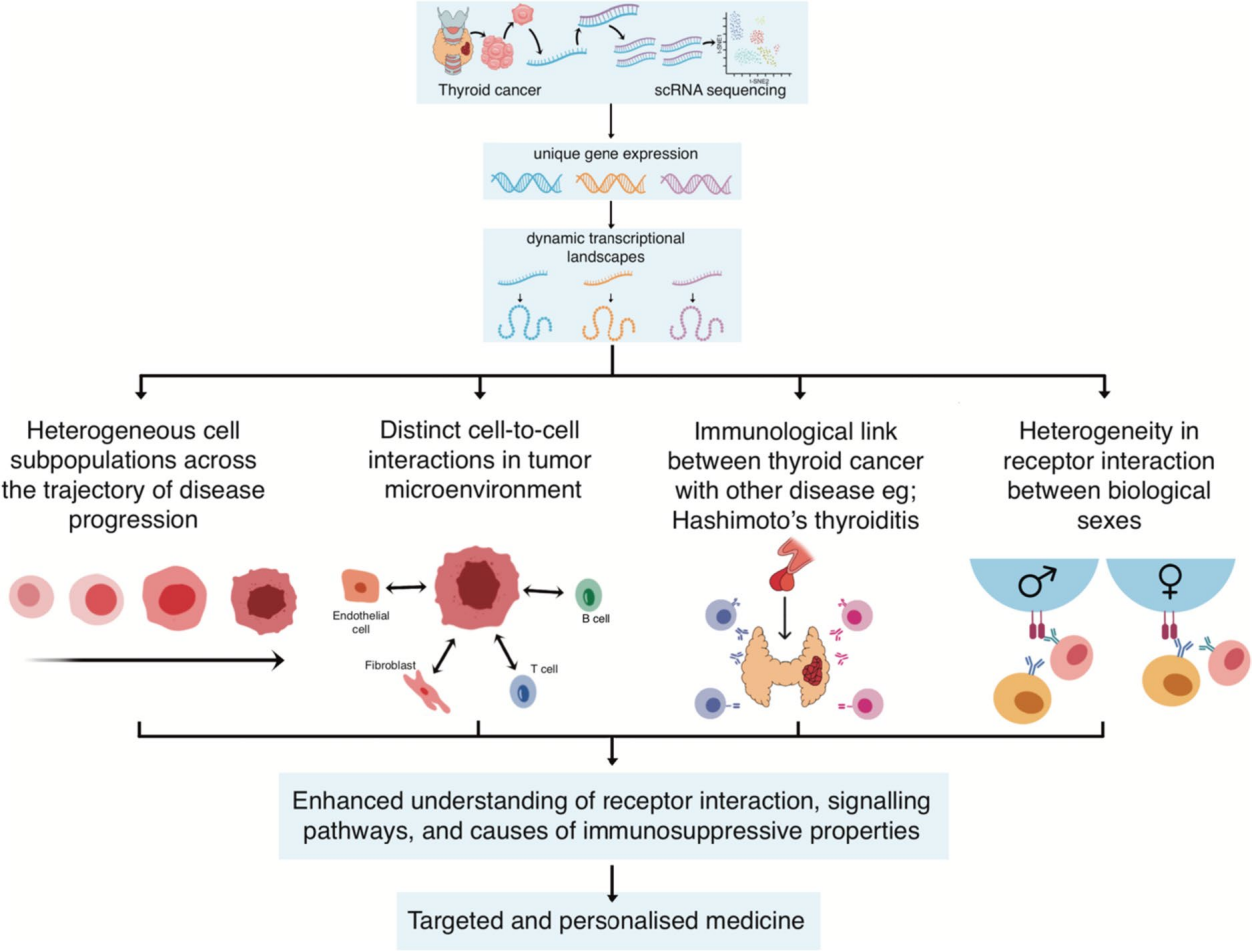
The role of single-cell RNA sequencing in cellular heterogeneity and tumour microenvironment in thyroid cancer. (Image originally created by authors) [1, 15, 20, 24–28]. *scRNA-seq* single-cell ribonucleic acid sequencing

Equally fascinating are the significant differences in the immune microenvironment between male and female PTC malignant epithelial cells that were uncovered with the help of scRNA-seq technology [28]. Regarding how supportive cells like fibroblasts and ECs interact with these malignant epithelial cells, females with PTC typically exhibit interactions involving human leukocyte antigen (HLA) family members and their receptors. On the other hand, males with PTC commonly showed involvement in transforming growth factor beta 1 (TGFB1) and its receptors [28]. Figure 2 depicts an overview of the role of scRNA-seq in cellular heterogeneity and TME in thyroid cancer.

### Novel biomarkers for therapeutic discovery

The identification of potential novel biomarkers is an essential step in guiding future research to understand their therapeutic implications. Recent studies employing scRNA-seq have contributed to a more comprehensive understanding of the TME of thyroid cancer. These studies have uncovered potential therapeutic targets and personalised immunotherapies and have supported the development of prognostic models for thyroid cancer.

Detailed analysis of scRNA-seq data from thyroid cancer cells has aided in the identification of novel biomarkers for PTC. Specifically, it has pointed out a critical LR pair biomarker, cell adhesion molecule 1 (CADM1)-CADM1, associated with a favourable prognosis in PTC [29]. Interestingly, the expression of CADM1-CADM1 was down-regulated in PTC, and overexpression of *CADM1* could inhibit tumour cell invasion and migration [29]. CADM1-CADM1 was more highly expressed in younger populations under 50 years old and stage I, compared to stage II and older populations over 50 years old [29]. Furthermore, a distinct *BRAF-*like B subtype with prominent dedifferentiation-like thyrocytes, enriched CAFs, and a worse prognosis was identified [20]. Additionally, vasculogenesis was found to be a critical characteristic of PTCs, with the tip EC phenotype suggested as a promising target for anti-angiogenic therapy (AAT), particularly anti-VEGFR antibodies, in thyroid cancer [20]. Together, these findings highlight CADM1-CADM1, the *BRAF*-like-B subtype, and the tip EC phenotype as potential prognostic and immunotherapeutic biomarkers for targeted treatment of PTC [20, 29].

For ATC, scRNA-seq has identified other novel biomarkers. These include CAFs, demonstrating strong interactions among mesenchymal cell types; macrophages, shifting from M1 to M2 states; and T cells, transitioning from cytotoxic to exhausted states [21]. ATC-specific immune checkpoint genes, including immunosuppressive molecules *VSIG4*, *LAIR1*, and *LILRB2*, were also identified [22]. The expression of *VSIG4* correlated significantly with tumour-infiltrating lymphocytes (B cells, CD8+ T cells, and Regulatory T cells (Tregs)) [22]. Also, the infiltration of interleukin 2 receptor subunit alpha (IL2RA)+ V-set and immunoglobulin domain-containing protein 4 (VSIG4)+ ATC-associated macrophages (ATAMs) showed a strong correlation with *BRAF* and *RAS* signalling and was associated with a favourable prognosis in thyroid cancer patients [22]. A potential functional role for *CREB3L1* in the dedifferentiation process and ATC development was also discovered [30]. These findings emphasise CAFs, macrophages, T cells, *VSIG4*, *LAIR1*, *LILRB2*, IL2RA+ VSIG4+ ATAMs, and *CREB3L1* as novel therapeutic targets that could inform personalised medicine for the treatment of ATC [21, 22, 30].

**Fig. 3:**
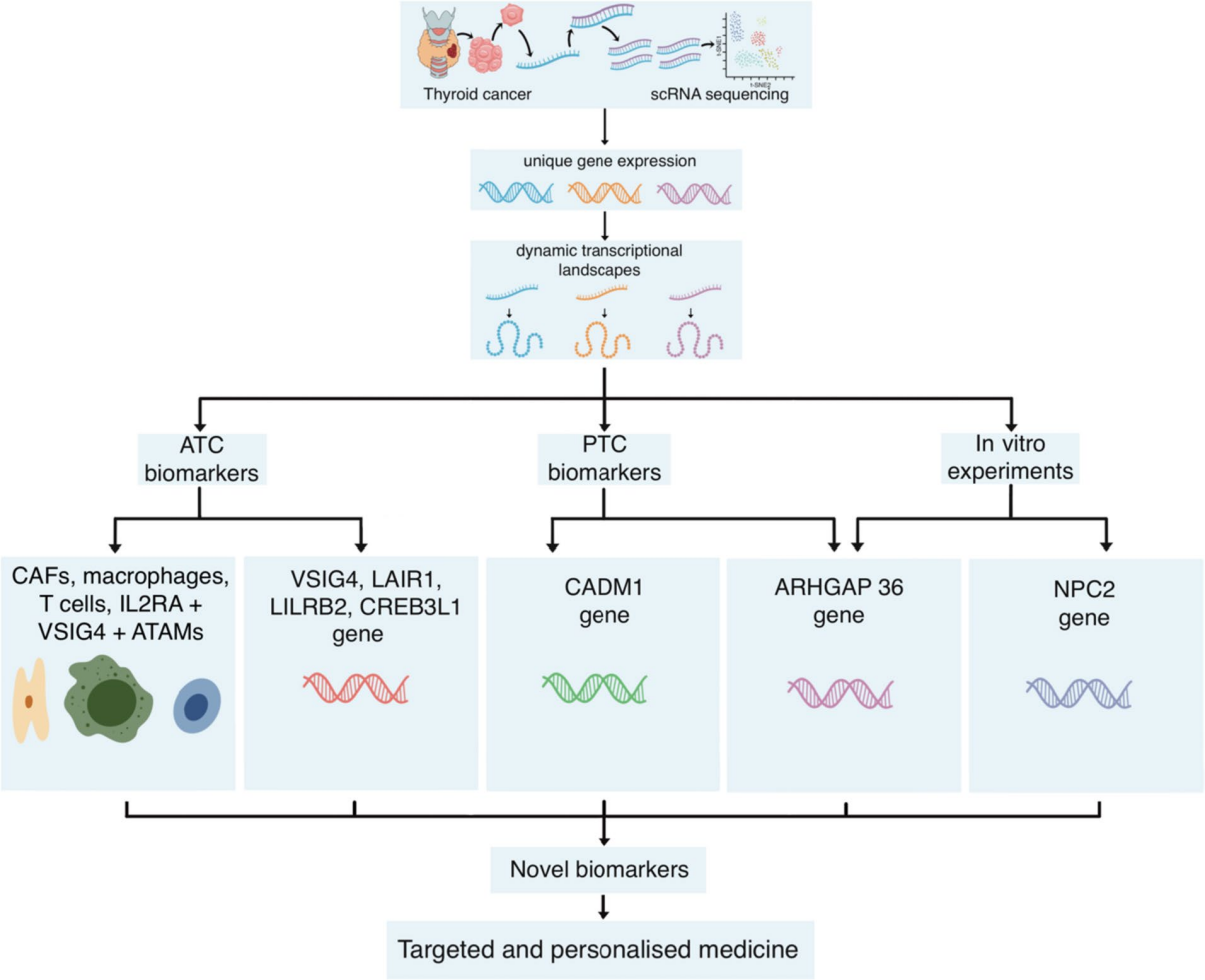
The role of single-cell RNA sequencing in identifying potential novel biomarkers of thyroid cancer. (Image originally created by authors) [20–22, 29–31]. *ATC* anaplastic thyroid carcinoma, *ATAMs* ATC-associated macrophages, *CAFs* cancer-associated fibroblasts, *IL2RA* interleukin 2 receptor subunit alpha, *PTC* papillary thyroid carcinoma, *scRNA-seq* single-cell ribonucleic acid sequencing, *VSIG4* V-set and immunoglobulin domain-containing protein 4

The integration of scRNA-seq with in vitro experiments has helped identify additional relevant biomarkers. For instance, the gene *ARHGAP36* was found to be expressed exclusively in the malignant cells of primary PTC and metastatic lesions, and it regulated their proliferation and migration [31]. Furthermore, the knockdown of *NPC2* was shown to significantly promote thyroid cancer cell apoptosis [1]. As a result, *NPC2* and *ARHGAP36* have been proposed as potential diagnostic and therapeutic targets for thyroid cancer [1, 31]. Figure 3 depicts the role of scRNA-seq in identifying potential novel biomarkers of thyroid cancers.

### Translational genomics: prognostic models, drug response, and mechanisms of resistance

Understanding drug response and resistance mechanisms is critical to designing effective treatment strategies and managing patient prognosis. ScRNA-seq has been used to elucidate the evolution of cell populations in progressive thyroid cancers, thereby providing important insights into disease progression dynamics [21]. This technique allows for the identification of novel cell subpopulations, the delineation of cellular hierarchies, and the discovery of rare cell types, all of which contribute to constructing prognostic models, predicting drug responses, and understanding drug resistance mechanisms.

Initial insights from scRNA-seq analyses have aided in constructing survival prognostic models through the identification of biomarkers. For instance, three genes*—FN1*, *CLU*, and *ANXA1*—that impact DNA and RNA methylation have been integrated into a prognostic model predicting reduced overall survival for high-risk patients [32]. Additionally, a six-gene prognostic signature—*CXCL3*, *CXCL1*, *IL1A*, *CCL5*, *TNFRSF12A*, and *IL18 —*within the cytokine receptor pathway in PTC has been identified, with increased risk scores associated with decreased overall survival [25]. A novel fibrosis score model, including six key differentially expressed fibroblast-related genes—*PCOLCE2*, *APOD*, *APOE*, *TIMP1*, *HTRA3*, and *MT1A,* was found to correlate with specific immune cell infiltration, leading to unfavourable clinical outcomes for thyroid cancer patients [15]. Prognostic models such as these hold significant promise in predicting clinical outcomes for thyroid cancer patients, thereby facilitating personalised medicine development and helping manage patient prognosis.

In order to translate these scientific discoveries into clinical practice and improve patient outcomes, understanding the mechanisms of drug response and resistance is crucial. For PTC patients with LN metastasis, scRNA-seq has revealed better immunotherapy targets than programmed cell death protein 1 (PD-1), such as T cell immunoreceptor with immunoglobulin and immunoreceptor tyrosine-based inhibitory motif domains (TIGIT) and CD96 [26]. Moreover, immune checkpoint genes have been found expressed in a small subset of Cytotoxic T cells in primary and metastatic PTCs, suggesting that targeting these genes may be effective for PTC therapy [24]. Notably, high expression of CADM1-CADM1 appears to significantly enhance the sensitivity to various targeted therapeutics, which could help overcome drug resistance [29]. Analysis of stem-like genes and differentiated cells in metastatic thyroid cancer suggested that regeneration of metastatic cancer might be driven by all persistent thyroid follicular epithelial cells [33]. These findings could guide the development of precision medicine, where therapeutic treatments are tailored to individual patients.

**Fig. 4:**
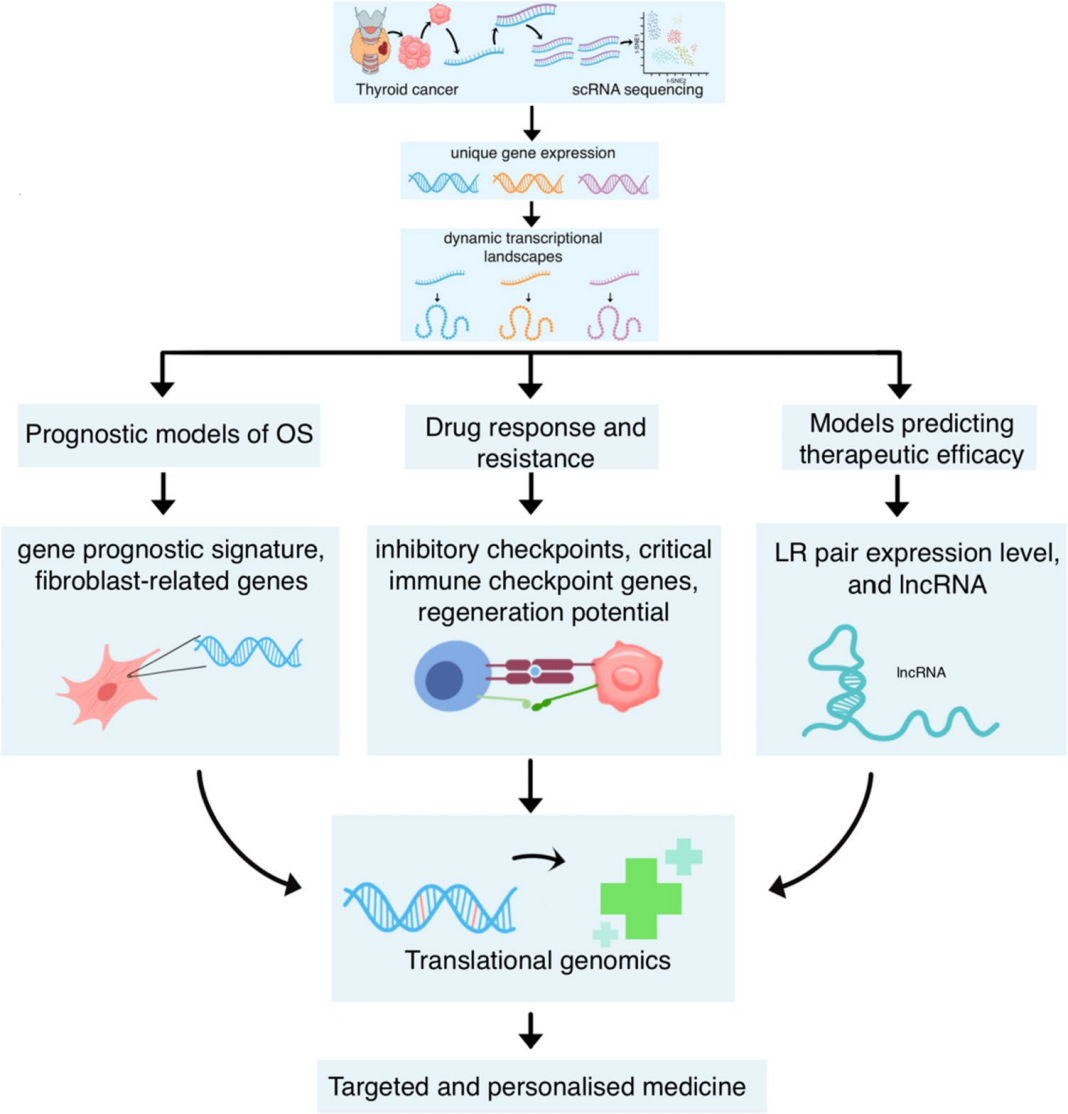
The role of single-cell RNA sequencing in thyroid cancer prognostic models, drug response, and resistance mechanisms. (Image originally created by authors) [15, 21, 24–26, 29, 32–35]. *mATC* mesenchymal ATC, *OS* overall survival, *scRNA-seq* single-cell ribonucleic acid sequencing

Finally, scRNA-seq can also assist in constructing models for accurate prediction of therapeutic efficacy. For instance, the construction of a risk model, LR score, was enabled by accurately identifying immune cell subsets’ characteristics. This model uses the LR pair expression level to predict PTC prognosis and immunotherapeutic response [34]. Another model uses six long non-coding RNAs (lncRNA) within thyroid cells at distinct stages of tumour progression. This long non-coding RNA signature has shown potential for prognosticating progression-free intervals and assessing the efficacy of RAI (I-131) therapy in PTC [35]. Such successful prediction models of therapeutic efficacy can enhance personalised medicine approaches and optimise healthcare resources by minimising expenditure on ineffective therapies. Figure 4 depicts the roles of scRNA-seq in thyroid cancer prognostic models, drug response, and resistance mechanisms.

## Challenges and limitations

### Technical challenges

Despite the transformative potential of scRNA-seq in the study of individual cells and exploring transcriptional heterogeneity within seemingly homogeneous cell populations, several challenges persist. The single-cell nature of scRNA-seq can complicate the task of assigning cell type or cell state to each sequenced cell [36]. This difficulty is particularly pronounced when identifying tumour cells within single-cell or spatial sequencing experiments [36].

Inherent in scRNA-seq data are issues of high dimensionality, technical noise, and sparsity, all requiring careful interpretation of results [37, 38]. Notably, technical noise tends to exhibit an inverse relationship with the quantity of starting material [37], thus presenting a significant challenge, especially when compared with bulk RNA-seq.

Moreover, scRNA-seq is susceptible to bias in amplification and losses in cDNA synthesis, both stemming from critical steps in the scRNA-seq protocol [39]. ‘Dropouts’, or instances where the transcriptome of each cell is incompletely captured, further compound the difficulties in analysing scRNA-seq data [40].

Finally, several studies involve limited sample sizes, which may only represent small subsets of the thyroid cancer population [20, 27, 32, 41]. Even though validation through public transcriptomic databases attempts to mitigate these effects, it would be advantageous to assess target biomarkers and gene expressions for a deeper understanding of tumour progression and thyroid cancer diversity in more extensive patient cohort studies or prospective investigations [20, 41].

#### Data analysis and interpretation

The successful application of scRNA-seq is dependent on accurate data analysis and interpretation. Nevertheless, contemporary scRNA-seq technology displays limitations that could impede data accuracy. For instance, scRNA-seq requires fresh samples rich in viable thyroid cancer cells, necessitating immediate analysis post-sample acquisition [20]. Consequently, scRNA-seq produces extensive datasets, frequently encompassing batch-specific systematic variations. This complexity poses a challenge for the effective removal of batch effects while preserving true biological characteristics associated with inter-tumour heterogeneities and data integration [20, 32, 42].

Moreover, achieving accurate genetic annotation of scRNA-seq datasets, such as differentiating thyrocyte clusters in thyroid cancer subtypes like PTC, remains a significant computational issue. This challenge is amplified by the complex, multi-layered identities or transitory states that these cell clusters may present [20, 43].

Further, the process of scRNA-seq separates cells from their native spatial context, a key determinant of cellular behaviour and fate [44]. This separation may hinder the ability to form comprehensive conclusions and potentially overlook important findings.

#### Financial and ethical challenges

The broader adoption and application of this technology in the field require addressing financial challenges and ethical considerations.

Several studies have underscored the significant costs involved in scRNA-seq experiments, including expenses on library preparation, reaction volumes, reagent costs, and computational resources [45–47]. These financial obstacles limit the accessibility of scRNA-seq technology, impeding its potential to further thyroid cancer research. Ethical concerns regarding health inequities may arise due to these elevated costs. Specifically, settings with limited resources, particularly low-and middle-income countries (LMICs), may struggle to afford this advanced technology, exacerbating disparities in health equity and research efforts.

Another pressing ethical concern involves the potential risks of downstream data linkage, which could lead to the re-identification of de-identified genetic data and questions surrounding patient understanding of their consent and the future use of their data [48]. While patients may consent to the risk of re-identification, further efforts are required to ensure patients understand the downstream application of their data within research initiatives.

Moreover, data from scRNA-seq, a next-generation sequencing (NGS) method, could not only pose privacy risks to individuals but also potentially raise concerns relating to groups and family members [48]. Research involving a small subset of a group could potentially be generalised to a larger group, leading to overgeneralization or, in more severe cases, stigma [48]. Certain members may feel their privacy has been compromised, fearing others might infer information about them based on their group affiliation [48].

Furthermore, unexpected findings from whole-genome analysis pose significant data privacy concerns [49]. It is important to secure genomic privacy and establish appropriate access to this data, especially when the initial consent does not encompass alternate studies [49].

**Fig. 5:**
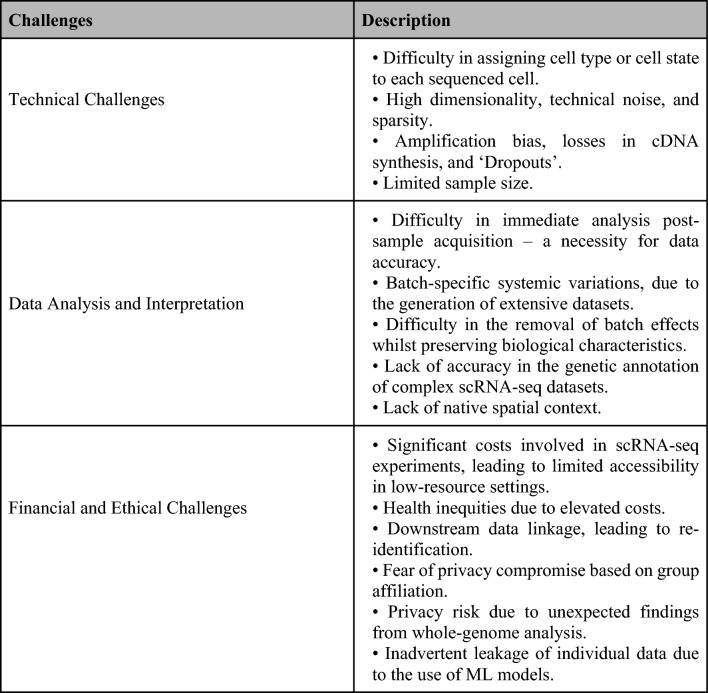
Summary of the challenges and limitations of single-cell RNA sequencing in thyroid cancer (Image originally created by authors) [20, 27, 32, 36–50]. *cDNA* complementary deoxyribonucleic acid, *ML* machine learning, *scRNA-seq* single-cell ribonucleic acid sequencing

Recent research suggests that machine learning (ML) models based on available data, including scRNA databases, may present specific risks. Models using public data or fewer parameters relative to subjects are generally considered less risky [50]. However, models configured with an extensive set of parameters and trained on individual-level genomic data or associated metadata may inadvertently disclose detailed information about study participants. This situation could potentially precipitate unforeseen privacy risks, requiring robust mechanisms to address such issues [50]. The details of the challenges of ScRNA-seq for thyroid cancers have been summarised in Fig. 5.

## Recent advances to improve ScRNA-seq and address its challenges

### Progress in computational methods

The integration of computational methods with scRNA-seq plays an important role in mitigating the difficulties posed by this technology. ScRNA-seq generates large datasets, often containing batch-specific systematic variations, complicating batch-effect removal and data integration [42]. Notably, Harmony, LIGER, and Seurat 3 have shown efficacy in batch integration while maintaining shorter runtimes [42]. Distinguishing subpopulations of cells across multiple datasets proves challenging. Yet, the application of the R toolkit Seurat enables general comparisons of scRNA-seq datasets, potentially enriching our understanding of how unique cell states respond to perturbation, disease, and evolution [51].

In the context of annotating scRNA-seq, the ‘FindAllMarkers’ function within Seurat offers a viable solution [21]. This technology enables the annotation of individual immune cell subclusters in thyroid cancer by facilitating differential gene expression analysis to identify significantly overexpressed genes within individual subclusters [21]. Moreover, adopting more flexible criteria to select additional signature genes for characterising thyroid cancer subclusters yielded improved annotation performance [21]. Using overlaps of signature genes with known markers from previous publications further aids in subcluster annotation [21].

Imputation methods have emerged to address systematic technical noise [52]. MAGIC, kNN-smoothing, and SAVER are among the recommended scRNA-seq imputation methods, showing superior performance compared to other methods [52]. Nonetheless, these methods exhibit significant variability in performance across aspects such as unsupervised clustering, pseudotemporal trajectory analyses, computational runtime, memory usage, and scalability [52]. Consequently, further studies are warranted to develop a more consistent method for addressing systemic technical noise in scRNA-seq data.

Lastly, to address the high costs associated with scRNA-seq, Cellular Indexing of Transcriptomes and Epitopes by Sequencing (CITE-seq) may be employed [46]. Optimising the antibody panel by removing antibodies unable to detect their target antigens and adjusting the concentrations of the remaining antibodies can improve analysis and potentially lower costs [46]. Such data could serve as a foundation for constructing an informative, cost-effective panel of CITE-Seq antibodies used at their optimal concentrations [46].

### The emergence of artificial intelligence, machine learning, and deep learning

The advent of artificial intelligence (AI) has revolutionised the implementation of scRNA-seq in thyroid cancer research. AI not only helps mitigate the inherent challenges of scRNA-seq but also amplifies its potential benefits.

The use of seven machine learning (ML) algorithms—namely ENet, Random Survival Forest (RSF), Ridge Regression, Support Vector Machine (SVM), StepCox, Gradient Boosting Machine (GBM), and Superpc—on disulfidptosis-related genes has shown promise in forecasting thyroid carcinoma patient prognosis [53]. In-depth analysis of disulfidptosis using scRNA-seq, ML, and the R package ‘oncoPredict’ has enabled the development of a novel classification system capable of effectively predicting the clinical prognosis and drug sensitivity of thyroid carcinoma patients [53]. Integration of the ML pipeline, Ikarus, may assist in assigning cell type or state to each sequenced cell in scRNA-seq. Ikarus has demonstrated proficiency in differentiating tumour cells from normal cells at the single-cell level with high sensitivity and specificity [36].

ML also proves useful in validating scRNA-seq results. scRNA analysis on PTC cells revealed that ‘PPARGi’ genes, consisting of 10 genes with a personalised prognostic index, are predominantly expressed in macrophages and epithelial cells [41]. ML algorithms demonstrated near-perfect performance of PPARGi in identifying the presence of the disease and in selecting a minor subset of patients with poor disease-specific survival rates in The Cancer Genome Atlas Thyroid Cancer Collection (TCGA-THCA) [41]. Moreover, ML facilitated the development of new merged microarray data (MMD) consisting exclusively of thyroid cancers and normal tissues [41].

ScRNA-seq data are characteristically high-dimensional, noisy, and sparse [38]. The advent of dimension reduction methods, such as the ML algorithm t-distributed stochastic neighbour embedding (t-SNE), has shown the highest accuracy and computational cost among several dimension reduction methods [38]. Additionally, devCellPy, an ML-enabled tool, allows for automated prediction of cell types across complex annotation hierarchies, species, and experimental systems with impressive accuracy and precision [43]. This facilitates a more accurate annotation of scRNA-seq datasets, where cells display complex, multi-layered identities or transitional states [43].

The single-cell Drug Response Analysis (scDEAL), another ML tool, employs a Domain-adaptive Neural Network (DaNN) to predict drug responses from scRNA-seq data, aiding in the study of cell programming, drug selection, and repurposing to improve therapeutic efficacy [54]. Moreover, uniform manifold approximation and projection (UMAP), a dimension reduction technique using a non-linear model and neural network, demonstrated superior stability, moderate accuracy, and the second highest computational cost among various dimension reduction methods [38].

The introduction of scDLC, a deep learning (DL) classifier, consistently outperforms existing methods like Poisson linear discriminant analysis (PLDA), negative binomial linear discriminant analysis (NBLDA), and zero-inflated PLDA (ZIPLDA) in classifying large scRNA-seq datasets [55].

ML and DL methods hold immense potential to enhance scRNA-seq technology, enabling effective prediction of clinical prognosis and drug sensitivity in thyroid cancer patients, as well as identifying the presence of PTC. Furthermore, the incorporation of ML helps navigate scRNA-seq challenges, including technical noise, loss of cell state or type, and high dimensionality. The future of ML applications in scRNA-seq appears promising.

### Single-cell data sharing and collaborative efforts

The growing interest in scRNA-seq presents challenges due to the technique’s high dimensionality and complexity [56]. To navigate these challenges, extensive collaborations between biologists, bioinformaticians, and biostatisticians are required [56]. The Single-Cell Transcriptomics Annotated Viewer (SCANNER) has emerged as a platform that enables convenient and collaborative scRNA-seq data sharing and analysis [56]. Equipped with a real-time database, SCANNER ensures secure data management and efficient exploration of gene set activation at the single-cell level [56].

The development of scDIOR allows for the transformation of single-cell data between the R and Python platforms [57]. This enables the linking of analytical tasks across these platforms, thereby simplifying the comparison of algorithm performance between them [57]. Critically, scDIOR accommodates a broad range of data types, including scRNA-seq and spatially resolved transcriptomics data, across programming languages and platforms at a swift pace [57]. This enables efficient and rapid analysis of scRNA-seq data.

Single Cell Explorer, a user-friendly Python-based web server application, offers another valuable tool. It empowers computational and experimental scientists to annotate cell expression phenotypes iteratively and collaboratively [58]. The application provides robust yet accessible features, such as identifying differential gene expression patterns for user-specified cell populations and facilitating cell type annotation using marker genes or differential gene expression patterns [58]. Additionally, scientists can employ the integrated web application, CellDepot, for quick upload, exploration, and comparison of scRNA-seq datasets, retaining information such as species and cell types [59]. This platform, when combined with the Cellxgene Visualisation Plugin (VIP) tool, offers refined insights such as cell composition and gene expression profiles, leveraging frequently applied plotting functions and high-level analysis methods [59]. This integration fosters a manageable and collaborative single-cell research community [59].

**Fig. 6:**
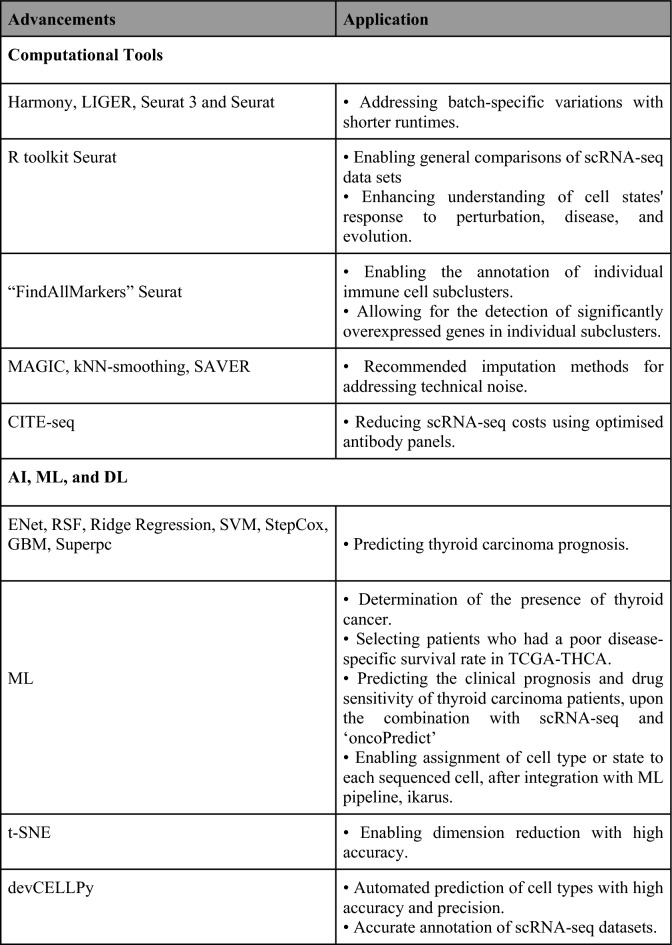

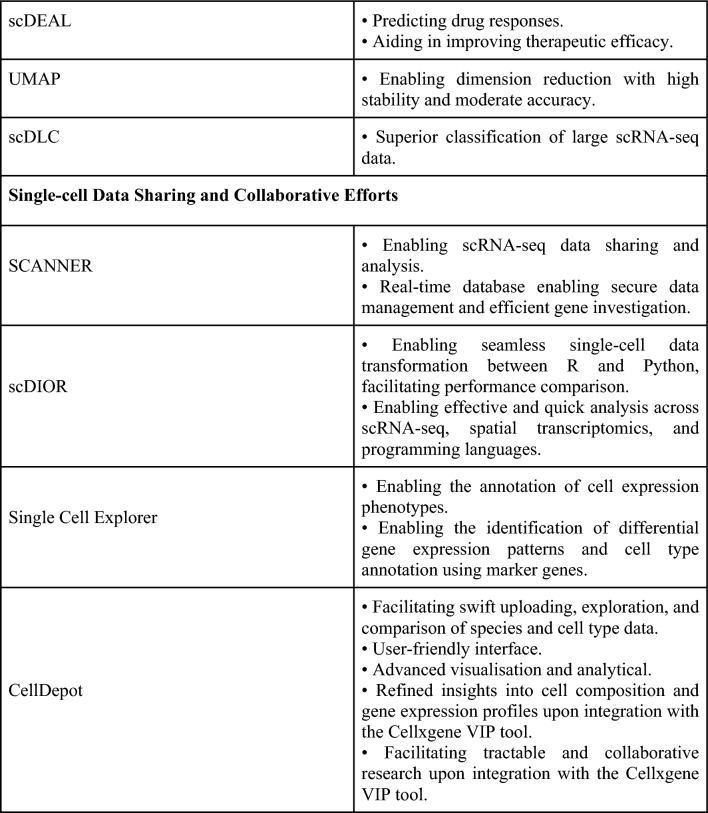
Recent advances in single-cell RNA sequencing techniques and their applications (Image originally created by authors) [21, 36, 38, 41–43, 46, 51–59]. *AI* artificial intelligence, *CITE-seq* cellular indexing of transcriptomes and epitopes by sequencing, *DL* deep learning, *ENet* efficient neural network, *GBM* gradient boosting machine, *RSF* random survival forest, *SCANNER* Single-Cell Transcriptomics Annotated Viewer, *scDEAL* single-cell drug response analysis, *scRNA-seq* single-cell ribonucleic acid sequencing, *StepCox* stepwise cox regression, *SVM* Support Vector Machine, *ML* machine learning, *t-SNE* t-distributed Stochastic Neighbour Embedding, *TCGA-THCA* The Cancer Genome Atlas Thyroid Cancer Collection, *t-SNE* t-distributed stochastic neighbour embedding, *UMAP* uniform manifold approximation and projection, *VIP* visualisation in plugin

Given the high dimensionality and complexity of scRNA-seq data, single-cell data sharing and collaborative efforts are of particular importance [56]. Recent advancements in scRNA-seq techniques and their applications are summarised in Fig. 6.

## Future directions

In recent years, ScRNA-seq has emerged as an innovative technique with significant potential for refining our understanding of thyroid cancer pathogenesis and potential treatment strategies. The studies considered in this review underline the capability of scRNA-seq to characterise thyroid cancer’s heterogeneity at single-cell resolution, discover novel tumour cell subpopulations, and provide insights into molecular mechanisms driving tumour progression. Despite these significant contributions, there remain several exciting avenues for future research to explore in the field of sc-RNA sequencing in thyroid cancer research.

A critical aspect of thyroid cancer requiring further exploration is intra-tumoral heterogeneity, which contributes to treatment resistance and recurrence. Future studies employing scRNA-seq should strive to fully elucidate the cellular diversity within individual tumours. Identifying unique cancer cell subpopulations and understanding their distinct gene expression profiles may pave the way for targeted therapeutic strategies, mitigating resistant clones, and preventing disease relapse [60]. Moreover, applying scRNA-seq to rare and under-characterised thyroid cancer subtypes is an area of significant interest. Investigating these less common malignancies at a single-cell resolution may yield invaluable insights into their unique biology and reveal potential vulnerabilities for targeted therapies [1].

While scRNA-seq has predominantly focused on protein-coding genes, the non-coding RNA landscape in thyroid cancer is largely underexplored. Future research should investigate the roles of lncRNAs, microRNAs, and other non-coding RNA species in thyroid cancer pathogenesis, as they may constitute important gene expression regulators and potential therapeutic targets [26].

The current findings from thyroid cancer cell scRNA-seq require validation through further in vivo and in vitro experimentation [20, 21, 29, 35]. In addition, future studies would be improved by functional validation of scRNA-seq findings, including iodine uptake and retention in various types of thyrocytes and the roles of identified TDS-associated genes in PTC tumorigenesis [20].

The establishment of ethical guidelines is critical to protecting patient confidentiality and ensuring researchers secure explicit consent from participants for the use of their data [48]. Moreover, careful consideration of potential result misinterpretation and unintended consequences is vital when applying scRNA-seq in thyroid cancer research. The ethical implications surrounding this technology highlight the necessity for an interdisciplinary approach, engaging clinicians, ethicists, and researchers to develop comprehensive frameworks supporting scientific rigour and ethical standards [48].

In order to ensure the affordability of scRNA-seq technology and prevent widening health equity gaps, future studies should focus on optimising cost-effective protocols [45]. Achieving cost-effectiveness for scRNA-seq involves multiple strategies, including technological innovations, workflow optimisations, and policy-level interventions. Innovative adjustments to existing techniques, such as the use of agarose microarrays and magnetic beads instead of oil droplet-coating samples [61], could help reduce costs while maintaining high output. The significant advancements yielded by scRNA-seq highlight the critical need for its evolution into a cost-effective healthcare solution, thus improving accessibility for healthcare providers and patients, particularly those grappling with socio-economic challenges in low-resource regions. The future prospects of sc-RNA-seq for thyroid cancer has been summarised in Fig. 4.

## Limitations of study

**Fig. 7:**
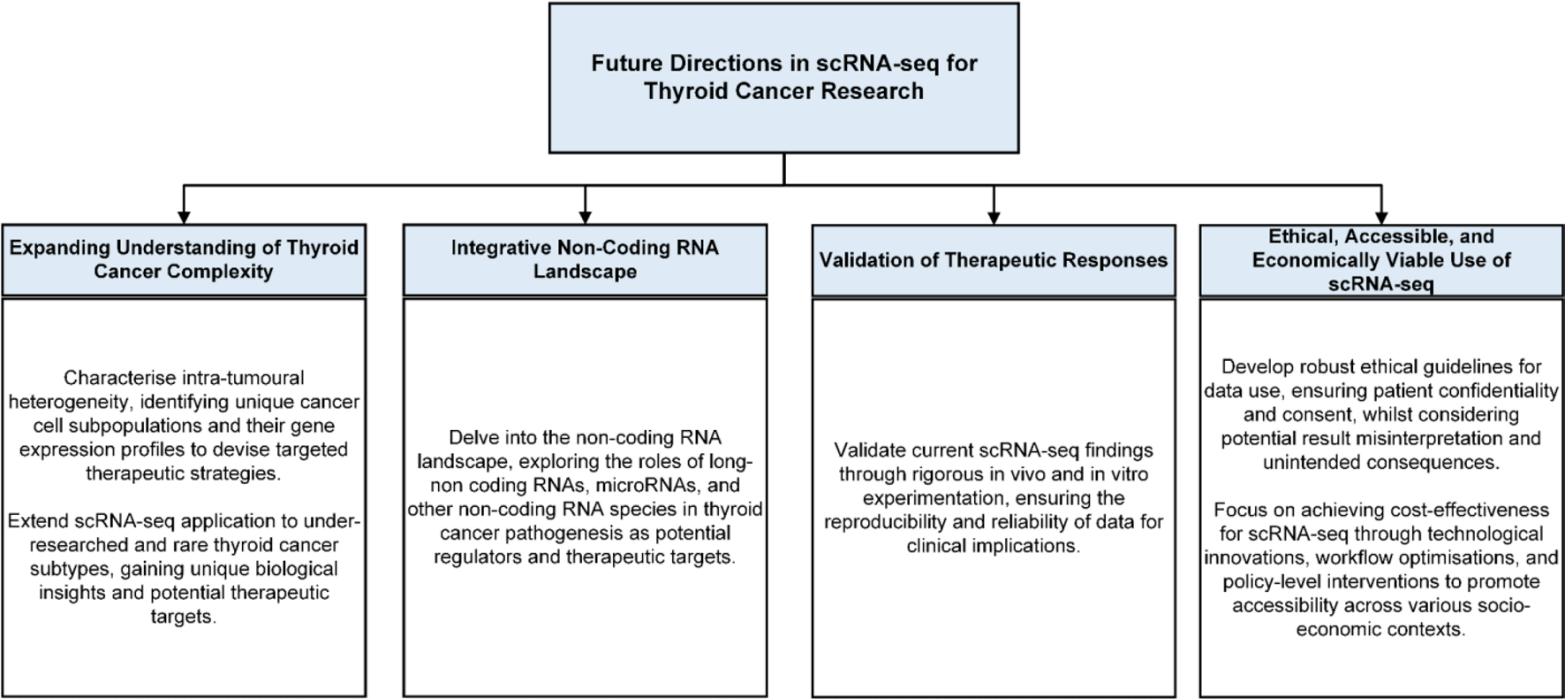
Future directions in single-cell rna-seq for thyroid cancer research (Image originally created by authors) [1, 20, 21, 26, 29, 35, 45, 48, 60, 61]. *scRNA-seq* single-cell ribonucleic acid sequencing, *RNA* ribonucleic acid

Despite the stringent methodology deployed in this study, several limitations need to be considered. Firstly, potential susceptibility to publication bias is a concern. There is a possibility that negative results may be underreported due to reputational concerns, while novel and impactful positive outcomes may be preferentially published. This could result in an unintentional bias in the literature (Fig. 7).

Secondly, language bias could be an additional limitation. Restricting the study to non-English literature might result in the inadvertent omission of relevant information, leading to potential knowledge gaps. Lastly, inherent biases associated with the processing of scRNA samples, such as batch effects [42], could distort interpretation and lead to inaccurate conclusions. These limitations underscore the importance of a careful and nuanced understanding of the existing literature when applying scRNA-seq to the study of thyroid cancers.

## Conclusion

The increasing incidence of thyroid cancer has underscored the significance of scRNA-seq for unravelling cellular heterogeneity, thereby enabling a thorough understanding of disease progression and the tailoring of treatment strategies. While the technique has its limitations, including potential biases and limitations in sample size, the rise of artificial intelligence and computational tools has significantly improved the interpretation of data. Future research directions should include the investigation of intra-tumoral heterogeneity, the integration of various ‘omics’ technologies, the exploration of non-coding RNA, and a focus on the study of rare thyroid cancer subtypes. Consequently, scRNA-seq could evolve into a cost-effective and powerful tool for the diagnosis, prognosis, and personalised treatment of thyroid cancer.

## Data Availability

Not Applicable.

## Abbreviations

AAT: Anti-angiogenic therapy
ACKR2: Atypical chemokine receptor 2
AI: Artificial intelligence
ATC: Anaplastic thyroid carcinoma
ATAMs: ATC-associated macrophages
CADM1: Cell adhesion molecule 1
CAFs: Cancer-associated fibroblasts
cDNA: Complementary deoxyribonucleic acid
CITE-seq: Cellular indexing of transcriptomes and epitopes by sequencing
CT: Computerised tomography
DaNN: Domain-adaptive Neural Network
DEFRG: Differentially expressed fibroblast-related genes
DL: Deep learning
DTC: Differentiated thyroid carcinoma
ECs: Endothelial cells
EGR1: Early growth response factor 1
ENet: Efficient Neural Network
GBM: Gradient boosting machine
HLA: Human leukocyte antigen
HT: Hashimoto’s thyroiditis
iATC: Inflammatory ATC
ICAM-1: Intercellular adhesion molecule-1
ICB: Immune checkpoint blockade
ICIs: Immune checkpoint inhibitors
IET: Iodine-exacerbated thyroiditis
IGF: Insulin-like growth factor
IL2RA: Interleukin 2 receptor subunit alpha
LMICs: Low-and middle-income countries
LN: Lymph node
lncRNAs: Long non-coding RNAs
LR: Ligand-receptor
mATC: Mesenchymal ATC
MCs: Mast cells
MFECs: Malignant follicular epithelial cells
MIF: Macrophage Migratory Inhibition Factor
ML: Machine learning
MMD: Merged microarray data
MRI: Magnetic resonance imaging
MTC: Medullary thyroid carcinoma
NBLDA: Negative binomial linear discriminant analysis
NFECs: Normal follicular epithelial cells
NGS: Next-generation sequencing
OS: Overall survival
PCa: Paracancerous tissue
PD-1: Programmed cell death protein 1
PFI: Progression-free intervals
PLDA: Poisson linear discriminant analysis
PTC: Papillary thyroid carcinoma
RAI: Radioactive iodine
RSF: Random Survival Forest
SCANNER: Single-Cell Transcriptomics Annotated Viewer
scDEAL: Single-cell drug response analysis
scRNA-seq: Single-cell ribonucleic acid sequencing
StepCox: Stepwise cox regression
SVM: Support Vector Machine
TCGA-THCA: The Cancer Genome Atlas Thyroid Cancer Collection
TCR: T cell receptor
TGFB1: Transforming growth factor beta 1
TIGIT: T cell immunoreceptor with immunoglobulin and immunoreceptor tyrosine-based inhibitory motif domains
TMB: Tumour mutation burden
TME: Tumour microenvironment
Tregs: Regulatory T cells
TSH: Thyroid-stimulating hormone
t-SNE: T-distributed stochastic neighbour embedding
UMAP: Uniform manifold approximation and projection
VIP: Visualisation in plugin
VEGFR: Vascular endothelial growth factor receptor
VSIG4: V-set and immunoglobulin domain-containing protein 4
ZIPLDA: Zero-inflated PLDA

## References

[1] Yang F (2023). Prognostic subtypes of thyroid cancer was constructed based on single cell and bulk-RNA sequencing data and verified its authenticity. Funct Integr Genomics.

[2] Pizzato M (2022). The epidemiological landscape of thyroid cancer worldwide: GLOBOCAN estimates for incidence and mortality rates in 2020. Lancet Diabetes Endocrinol.

[3] Cramer JD (2010). Analysis of the rising incidence of thyroid cancer using the Surveillance, Epidemiology and End Results national cancer data registry. Surgery.

[4] Baker SR, Bhatti WA (2006). The thyroid cancer epidemic: is it the dark side of the CT revolution?. Eur J Radiol.

[5] Pellegriti G, et al. Worldwide increasing incidence of thyroid cancer: update on epidemiology and risk factors. J Cancer Epidemiol. 2013;965212. 10.1155/2013/965212 PMC3664492 23737785

[6] Tsatsoulis A (2018). The role of insulin resistance/hyperinsulinism on the rising trend of thyroid and adrenal nodular disease in the current environment. J Clin Med.

[7] Araque KA, Gubbi S, Klubo-Gwiezdzinska J (2020). Updates on the management of thyroid cancer. Horm Metab Res.

[8] Maniakas A (2020). Evaluation of overall survival in patients with anaplastic thyroid carcinoma, 2000–2019. JAMA Oncol.

[9] National Cancer Institute. All Cancer Sites Combined Recent Trends in SEER Age-Adjusted Incidence Rates, 2000–2020. Seer*explorer application. 2022. https://seer.cancer.gov/statistics-network/explorer/application.html Accessed 30 July 2023.

[10] Sosa JA (2013). Increases in thyroid nodule fine-needle aspirations, operations, and diagnoses of thyroid cancer in the United States. Surgery.

[11] Ravetto C, Colombo L, Dottorini ME (2000). Usefulness of fine-needle aspiration in the diagnosis of thyroid carcinoma: a retrospective study in 37,895 patients. Cancer.

[12] Udelsman R (2001). Randomized prospective evaluation of frozen-section analysis for follicular neoplasms of the thyroid. Ann Surg.

[13] Steele CB (2017). Vital signs: trends in incidence of cancers associated with overweight and obesity—United States, 2005–2014. Morb Mortal Wkly Rep..

[14] Karzai S (2022). Ambient particulate matter air pollution is associated with increased risk of papillary thyroid cancer. Surgery.

[15] Li W (2022). Integrated analysis of fibroblasts molecular features in papillary thyroid cancer combining single-cell and bulk RNA sequencing technology. Front Endocrinol.

[16] Jovic D (2022). Single-cell RNA sequencing technologies and applications: a brief overview. Clin Transl Med.

[17] Wang Y (2020). Advances of droplet-based microfluidics in drug discovery. Expert Opin Drug Discov.

[18] Adil A (2021). Single-cell transcriptomics: current methods and challenges in data acquisition and analysis. Front Neurosci.

[19] Van de Sande B (2023). Applications of single-cell RNA sequencing in drug discovery and development. Nat Rev Drug Discov.

[20] Pu W (2021). Single-cell transcriptomic analysis of the tumor ecosystems underlying initiation and progression of papillary thyroid carcinoma. Nat Commun.

[21] Lu L (2023). Anaplastic transformation in thyroid cancer revealed by single-cell transcriptomics. J Clin Investig.

[22] Pan Z (2023). IL2RA+VSIG4+ tumor-associated macrophage is a key subpopulation of the immunosuppressive microenvironment in anaplastic thyroid cancer. Biochim Biophys Acta Mol Basis Dis.

[23] Amanullah M (2023). Tumor-infiltrating immune cell landscapes in the lymph node metastasis of papillary thyroid cancer. Curr Oncol.

[24] Yan T (2021). Single-cell transcriptomic analysis of ecosystems in papillary thyroid carcinoma progression. Front Endocrinol.

[25] Wang T (2022). Single-cell transcriptome analysis reveals inter-tumor heterogeneity in bilateral papillary thyroid carcinoma. Front Immunol.

[26] Wang Z (2021). Single-cell RNA sequencing reveals a novel cell type and immunotherapeutic targets in papillary thyroid cancer. medRxiv.

[27] Pan J (2021). Papillary thyroid carcinoma landscape and its immunological link with hashimoto thyroiditis at single-cell resolution. Front Cell Dev Biol.

[28] Peng M (2021). Single-cell transcriptomic landscape reveals the differences in cell differentiation and immune microenvironment of papillary thyroid carcinoma between genders. Cell Biosci.

[29] He H (2022). Analysis of the key ligand receptor CADM1_CADM1 in the regulation of thyroid cancer based on scRNA-seq and bulk RNA-seq data. Front Endocrinol.

[30] Luo H (2021). Characterizing dedifferentiation of thyroid cancer by integrated analysis. Sci Adv.

[31] Yan T (2021). ARHGAP36 regulates proliferation and migration in papillary thyroid carcinoma cells. J Mol Endocrinol.

[32] Chen Z (2022). Single-cell rna sequencing revealed a 3-gene panel predicted the diagnosis and prognosis of thyroid papillary carcinoma and associated with tumor immune microenvironment. Front Oncol.

[33] Hu A (2020). Single-cell RNA sequencing reveals the regenerative potential of thyroid follicular epithelial cells in metastatic thyroid carcinoma. Biochem Biophys Res Commun.

[34] Cao ZX (2022). Receptor-ligand pair typing and prognostic risk model for papillary thyroid carcinoma based on single-cell sequencing. Front Immunol.

[35] Hao J (2023). A novel autophagy-related long non-coding RNAs signature predicting progression-free interval and I-131 therapy benefits in papillary thyroid carcinoma. Open Med.

[36] Dohmen J (2022). Identifying tumor cells at the single-cell level using machine learning. Genome Biol.

[37] Brennecke P (2013). Accounting for technical noise in single-cell RNA-seq experiments. Nat Methods.

[38] Xiang R (2021). A comparison for dimensionality reduction methods of single-cell RNA-seq data. Front Genet.

[39] Islam S (2014). Quantitative single-cell RNA-seq with unique molecular identifiers. Nat Methods.

[40] Qiu P (2020). Embracing the dropouts in single-cell RNA-seq analysis. Nat Commun.

[41] Kim J (2021). PPARγ targets-derived diagnostic and prognostic index for papillary thyroid cancer. Cancers.

[42] Tran HTN (2020). A benchmark of batch-effect correction methods for single-cell RNA sequencing data. Genome Biol.

[43] Galdos FX (2022). devCellPy is a machine learning-enabled pipeline for automated annotation of complex multilayered single-cell transcriptomic data. Nat Commun.

[44] Satija R (2015). Spatial reconstruction of single-cell gene expression data. Nat Biotechnol.

[45] Chen W (2017). Profiling of single-cell transcriptomes. Curr Protoc Mouse Biol..

[46] Nettersheim FS (2022). Titration of 124 antibodies using CITE-Seq on human PBMCs. Sci Rep.

[47] Haque A (2017). A practical guide to single-cell RNA-sequencing for biomedical research and clinical applications. Genome Med.

[48] Martinez-Martin N, Magnus D (2019). Privacy and ethical challenges in next-generation sequencing. Expert Rev Precis Med Drug Dev.

[49] Ong FS, Grody WW, Deignan JL (2011). Privacy and data management in the era of massively parallel next-generation sequencing. Expert Rev Mol Diagn.

[50] Byrd JB (2020). Responsible, practical genomic data sharing that accelerates research. Nat Rev Genet.

[51] Butler A (2018). Integrating single-cell transcriptomic data across different conditions, technologies, and species. Nat Biotechnol.

[52] Hou W (2020). A systematic evaluation of single-cell RNA-sequencing imputation methods. Genome Biol.

[53] Feng Z (2023). Identification a unique disulfidptosis classification regarding prognosis and immune landscapes in thyroid carcinoma and providing therapeutic strategies. J Cancer Res Clin Oncol.

[54] Chen J (2022). Deep transfer learning of cancer drug responses by integrating bulk and single-cell RNA-seq data. Nat Commun.

[55] Zhou Y (2022). scDLC: a deep learning framework to classify large sample single-cell RNA-seq data. BMC Genomics..

[56] Cai G (2022). SCANNER: a web platform for annotation, visualization and sharing of single cell RNA-seq data. Database..

[57] Feng H, Lin L, Chen J (2022). scDIOR: single cell RNA-seq data IO software. BMC Bioinform.

[58] Feng D (2019). Single Cell Explorer, collaboration-driven tools to leverage large-scale single cell RNA-seq data. BMC Genomics.

[59] Lin D (2021). Cell depot: a unified repository for scRNA-seq data and visual exploration. J Mol Biol.

[60] Li P-H (2022). Recent developments in application of single-cell RNA sequencing in the tumour immune microenvironment and cancer therapy. Mil Med Res.

[61] Han X (2018). Mapping the mouse cell atlas by microwell-seq. Cell.

