# Correction to: Exploring the advances of single‑cell RNA sequencing in thyroid cancer: a narrative review

**DOI:** 10.1007/s12032-024-02304-w

**Published:** 2024-02-23

**Authors:** Joecelyn Kirani Tan, Wireko Andrew Awuah, Sakshi Roy, Tomas Ferreira, Arjun Ahluwalia, Saibaba Guggilapu, Mahnoor Javed, Muhammad Mikail Athif Zhafir Asyura, Favour Tope Adebusoye, Krishna Ramamoorthy, Emma Paoletti, Toufik Abdul-Rahman, Olha Prykhodko, Denys Ovechkin

**Affiliations:** 1 Faculty of Medicine, University of St Andrews, St Andrews, Scotland, UK; 2 Faculty of Medicine, Sumy State University, Sumy, Ukraine; 3 School of Medicine, Queen’s University Belfast, Belfast, UK; 4 School of Clinical Medicine, University of Cambridge, Cambridge, UK; 5 Faculty of Medicine, Bangalore Medical College and Research Institute, Bengaluru, India; 6 School of Medicine, The University of Nottingham, Nottingham, NG7 2UH UK; 7 Faculty of Medicine, Universitas Indonesia, Jl. Salemba Raya No.6, Jakarta, 10430 Indonesia; 8 Rutgers University-New Brunswick, New Brunswick, NJ 08854 USA; 9 Faculty of Medicine, University of Manchester, Manchester, M13 9WJ UK

**Correction to: Medical Oncology (2024) 41:27** 10.1007/s12032-023-02260-x

The original version of this article contained a mistake in one of the co-author's first name. The co-author’s name should read as "Olha Prykhodko" instead of "Olga Prykhodko". It is now corrected with this correction.

The original article has been corrected.

